# The SCM test for cancer. an evaluation in terms of lymphocytes from healthy donors and cancer patients.

**DOI:** 10.1038/bjc.1980.140

**Published:** 1980-05

**Authors:** H. Mitchell, P. Wood, C. R. Pentycross, E. Abel, K. D. Bagshawe

## Abstract

The SCM test was established as originally described, and an attempt was made to evaluate it using myelin basic proteins. Various later modifications described by the original authors were incorporated as they were communicated to us. In separate studies attempts were also made to overcome some of the problems which seemed inherent in the technique. In the small series for which valid results were obtained we were unable to confirm the original claim that the method discriminates between cancer patient lymphocytes and those from non-cancer subjects with almost 99% reliability. Indeed, although differences were found between the mean SCM values of cancer patients and of healthy controls, these differences were not significant.


*Br. J. Cancer* (1980) **41**, 772

### THE SCM TEST FOR CANCER. AN EVALUATION IN TERMS OF LYMPHOCYTES FROM HEALTHY DONORS AND CANCER PATIENTS

H. MITCHELL, P. WOOD*, C. R. PENTYCROSS, E. ABEL AND K. D. BAGSHAWE

*From the Department of Medical Oncology, Charing Cross Hospital, Fulham Palace Road, London W6, and *St Mary's Hospital, Paddington, London W2*

Received 10 September 1979 Accepted 10 December 1979

**Summary.—The SCM test was established as originally described, and an attempt was made to evaluate it using myelin basic proteins. Various later modifications described by the original authors were incorporated as they were communicated to us. In separate studies attempts were also made to overcome some of the problems which seemed inherent in the technique. In the small series for which valid results were obtained we were unable to confirm the original claim that the method discriminates between cancer patient lymphocytes and those from non-cancer subjects with almost 99% reliability. Indeed, although differences were found between the mean SCM values of cancer patients and of healthy controls, these differences were not significant.**

THE "Structuredness of Cytoplasmic Matrix" (SCM) test was reported by Cercek *et al.* (1974) as a means of distinguishing between cancer patients and normal subjects on the basis of changes in fluorescence polarization (p) of lymphocytes following incubation with Cancer Basic Protein (CaBP), Encephalitogenic Factor (EF) and Phytohaemagglutinin (PHA). In lymphocytes from cancer patients a decrease in p value (compared to the control) was observed after incubation with CaBP or EF, but not with PHA. Conversely in lymphocytes from healthy individuals, the p value decreased with PHA, but not with CaBP or EF. The ratio of the CaBP response to the PHA response gave a value known as the SCM response ratio ($RR_{SCM}$) by means of which a confident prediction of the donor's cancer status could be made. In this paper we relate our experience in trying to confirm these observations.

During the course of a Workshop held in November 1976 and subsequently published (Bagshawe, 1977) a revised technical protocol elucidating several crucial points was presented. It was stated that a graph of the fluorescein emission-polarization spectrum for "resting state" lymphocytes revealed a peak at 510 nm which disappeared after stimulation of the cells.

This observation was fundamental to the measurement of p-value changes in the SCM test and invalidated any approach which used different light sources or monochromators, a different arrangement of polarizing filters or lenses or different substrate conditions from those specified. A further modification related to the Ficoll–Triosil used for the preparation of lymphocytes. A specific gravity at 25°C of 1·081 g/cm³ and osmolality of 320 mOsm/kg was necessary to achieve a distinctive two-layer separation of lymphocytes at the plasma-gradient interface with the SCM-responding cells present only in the upper layer, or the upper portion if only one layer was produced. The correct pH (7·4) and calcium-ion concentration of the phosphate-buffered saline (PBS) were also critical.

In a later review (Cercek & Cercek, 1977) these parameters were again described and a modification whereby the substrate was prepared to a final concentration of $6{\cdot}25 \times 10^{-7}$M from a stock solution in acetic acid rather than acetone was also suggested.

We present results of SCM tests performed in this laboratory, details of the cells isolated, and studies on the intracellular fluorescein.

### MATERIALS AND METHODS

Initially the SCM tests were performed on a Perkin-Elmer model MPF-3 spectrofluorimeter, but the majority of the work was on a model MPF-4, following the protocol as originally described (Cercek *et al.*, 1974) after adoption of the various modifications described at the 1976 Workshop. We have also tried, as far as possible, to keep abreast of the alterations in technique as they were communicated to us.

Reagent-grade PHA was obtained from Wellcome Reagents Ltd. Myelin basic protein (MBP) was prepared using an established technique (Deibler *et al.*, 1972).

Thymus-dependent "T" cells and bursa-equivalent "B" cells were assessed by a modification of a published rosetting technique (Biozzi *et al.*, 1968).

Verification of the fluorescein emission-polarization spectrum was attempted in a number of ways. Firstly, by measuring p values on the MPF-4 with the emission slit width set at 5 nm, the emission wavelength was varied in 5nm steps from 500 nm up to 550 nm. Secondly, p values were measured on lymphocytes which had been incubated with substrate and then spun to produce a pellet of cells in a strain-free glass capillary, which was then analysed spectrally. Finally, cells under a Leitz microscope were illuminated with incident Xenon light *via* a light guide, and the individual cell fluorescence conducted *via* another light guide to be analysed on the MPF-4.

The kinetics of substrate uptake and intracellular fluorescein production were determined on $5 \times 10^7$ lymphocytes uniformly suspended in 100 ml of stirred substrate at 27°C. The chart recorder was started when the cells were added and, at about 30-s intervals, a 3-ml aliquot was removed, placed in a cuvette, and the total fluorescence recorded. The sample was then filtered through a Millipore multifiltration (24 place) manifold and the filtration time noted. The filtrates were measured at the end of the experiment and the values recorded on the chart. The cellular fluorescein was derived by the conventional subtraction of the filtrate value from the total fluorescence, and also directly by lysis of the cells retained on the Millipore membrane. Treatment of the lysate with strong alkali further provided a measure of the unhydrolysed intracellular substrate.

A simple device was constructed consisting of a Zinc lamp parallel light source and 2 side-window photomultiplier tubes mounted on either side of the thermostatically controlled cuvette. The fluorescein emission was simultaneously analysed with film polarizers and barrier filters to produce 2 signals corresponding to the emission intensities parallel and perpendicular to the exciting light source. These were both displayed on a twin-pen chart recorder and processed electronically to produce a continuous read-out of p. The apparatus was demonstrated at the Workshop in 1976.

### RESULTS

The p values of unstimulated lymphocytes and after incubation with either PHA or MPB are shown in Table I for the original technique and for the post-Workshop protocol. As can be seen, the

TABLE I.—*Changes in mean values of p obtained in our laboratory before and after modifications of technique introduced following the* 1976 *Workshop*

| | Normal | | | Cancer | | | |
|---|---|---|---|---|---|---|---|
| | Mean p | % C of V | Δ % p | Mean p | % C of V | Δ % p | Average |
| *Pre-Workshop* | | | | | | | |
| Control | 0·136 | 4·3 | — | 0·145 | 11·7 | — | 0·141 |
| PHA | 0·137 | 14·3 | 0 | 0·125 | 18·5 | −14·1 | 0·131 |
| MBP | 0·137 | 8·0 | 0 | 0·134 | 10·0 | −7·6 | 0·136 |
| *Post-Workshop* | | | | | | | |
| Control | 0·158 | 11·9 | — | 0·157 | 12·3 | — | 0·158 |
| PHA | 0·136 | 9·4 | −13·7 | 0·148 | 9·1 | −6·2 | 0·142 |
| MBP | 0·150 | 9·3 | −4·6 | 0·140 | 15·1 | −11·0 | 0·145 |

FIG. 1.—% changes of p (fluorescence polarization) in lymphocytes from non-cancer and cancer subjects following incubation with phytohaemagglutinin (PHA) and myelin basic protein (MBP). Bars show mean and s.d.

FIG. 2.—SCM response ratios ($RR_{SCM}$) of non-cancer and cancer subjects. Individual values plus mean and s.d.

mean p of unstimulated lymphocytes increased by 12·1% from 0·141 to 0·158. This increase in p may correspond to the use of the higher osmolality PBS (330 mOsm) introduced after the Workshop, compared to the original Dulbecco complete PBS (280 mOsm). These values are lower than the mean value of 0·206 ± 0·002 (s.e.) reported by Cercek *et al.* (1974) or 0·196 ± 0·004 reported by Cercek & Cercek (1977) for healthy control lymphocytes. It does, however, fall within 1 s.d. of the latter value (range 0·130 to 0·262) and is comparable to the values reported by M. Stack-Dunne (0·153 ± 0·002 and 0·142 ± 0·003) and J. P. Dickinson (0·160 ± 0·020) at the November 1976 Workshop. Dickinson also found a 13% increase in mean p for unstimulated lymphocytes after incorporation of the Workshop recommendations, as well as significant improvement in the response of healthy control lymphocytes to PHA (personal communication).

Whereas there was little response to either PHA or MBP shown by healthy control lymphocytes using the original technique, a decrease of 13·7% with PHA and 4·6% with MBP was observed using the revised protocol. For lymphocytes from cancer subjects, the response to PHA incubation changed from a decrease of 14·1% to a decrease of 6·2%, whereas the response to MBP improved from a decrease of 7·6% to a decrease of 11·0%. Although these results taken together gave an $RR_{SCM}$ of 1·06 for healthy individuals and 0·99 for cancer patients, the spread of results for individual samples is considerable, as shown in Fig. 1 (% changes in p) and Fig. 2 ($RR_{SCM}$). Only 60% of either healthy controls or cancer subjects could be considered to give the "correct" $RR_{SCM}$ response, frequently because a change in p was noted in both PHA- and MBP-incubated lymphocytes.

An assessment of the cells found in the 2 layers was made using blood from 15 normals and 15 cancer subjects.

Simple microscopic examination, cell count and differential count performed on samples from both healthy individuals and cancer patients revealed that the lower layers were frequently contaminated by clumped erythrocytes, as well as polymorphonuclear cells (PMN) which presumably failed to ingest iron particles at the first stage of preparation. Lymphocyte purity in each layer is summarized in Table II. The differences between the 2 layers are significant. Each layer was also

TABLE II.—*Purity of lymphocyte suspensions (% lymphocytes) in each layer*

| Normal subjects (15) | | Cancer patients (15) | |
|---|---|---|---|
| Upper layer | Lower layer | Upper layer | Lower layer |
| Range | Range | Range | Range |
| 92–99 | 75–95 | 92–99 | 78–92 |
| Mean | Mean | Mean | Mean |
| 95·3 | 85·3 | 95·2 | 83·7 |
| $P<0{\cdot}05$ | | $P<0{\cdot}01$ | |

TABLE III.—*E-rosettable lymphocytes (%) in each layer*

| Normal subjects (15) | | Cancer patients (15) | |
|---|---|---|---|
| Upper layer | Lower layer | Upper layer | Lower layer |
| Range | Range | Range | Range |
| 30·1–53·3 | 20·1–48·8 | 36·2–51·7 | 32·5–50·3 |
| Mean | Mean | Mean | Mean |
| 44·2 | 40·8 | 43·9 | 40·5 |
| $P<0{\cdot}01$ | | $P<0{\cdot}05$ | |

TABLE IV.—*EAC-rosettable lymphocytes (%) in each layer*

| Normal subjects (15) | | Cancer patients (15) | |
|---|---|---|---|
| Upper layer | Lower layer | Upper layer | Lower layer |
| Range | Range | Range | Range |
| 7·4–16·2 | 7·2–21·1 | 6·6–19·5 | 9·6–21·6 |
| Mean | Mean | Mean | Mean |
| 10·8 | 13·9 | 11·9 | 16·3 |
| $P<0{\cdot}05$ | | $P<0{\cdot}01$ | |

assessed in terms of T and B cells by the rosetting technique. The results in Tables III and IV show that the ratio of T cells (E rosetting) to B cells (EAC rosetting) is significantly greater in the upper-layer lymphocytes.

The fluorescein emission-polarization spectrum for a number of different lymphocytes revealed a broad plateau around 525 nm, without the described sharp peak at 510 nm. In addition, the studies on the packed cells showed a pronounced "Red Shift" ($\lambda_{max}=527$ nm), indicating an intracellular fluorescein concentration of greater than $10^{-5}$M. This was confirmed microscopically, despite rapid photobleaching.

The results of the multifiltration experiments are displayed in Fig. 3 and show that the intracellular fluorescein concentration levels out after about 10 min and that the polarization decreases with the increasing cellular level, which is in agreement with findings already published (Preece *et al.*, 1978).

Direct measurement of cell lysates indicated that the intracellular FDA reached a maximum within 30 s and that the cellular fluorescein was considerably in excess of the value calculated by subtraction of filtrate from total fluorescein. This suggests that significant quenching of fluorescein is occurring within the cell.

Results obtained on the direct polarization instrument showed p to remain constant for several minutes at the start of the reaction, and then to reduce as the fluorescein released from the cells accumulated in the background. The lymphocyte p values obtained by this direct method were somewhat lower than those obtained on the MPF-4, averaging 0·130.

FIG. 3.—Broken line—change of p with time. Solid lines—increase in fluorescein concentrations with time. Total concentration refers to the total fluorescence output ($I_{\parallel}+2I_{\perp}$) from the cell and substrate suspension.

### DISCUSSION

The phenomenon of fluorescence is initiated by the interaction of a photon of exciting light with the electron cloud of a suitably oriented fluorescent probe molecule. There is then a rearrangement of the quantum of energy within the molecule which takes a length of time known as tau ($\tau$) during which the molecule may rotate. Finally, a photon is ejected at a longer wavelength, since there is an energy loss during the process, and this photon is polarized in the same plane relative to the molecule as the original photon which was absorbed. It follows that analysis of the plane of the polarization of the light emitted following excitation with polarized light indicates by how much the molecules have rotated during the time, $\tau$. In highly viscous media, the molecules can rotate little and the emitted light will nearly all be parallel to the exciting light. In very low-viscosity media, the molecules will have rotated to a state of randomness before emission, therefore analysis will show the photons to be equally distributed, parallel and perpendicular to the exciting light.

Fluorescence-polarization measurement is a very useful tool for the study of the environment in which the probe is located and a wide variety of different compounds have been used to study different regions of the cell.

In our study, although the population was far from ideal in terms of non-malignant controls, age-matching or numbers, significant discrimination between healthy controls and cancer subjects should have been expected on the basis of the published techniques.

Whilst there was some improvement in response to PHA or MBP using the revised protocol, the "correct" diagnosis of only 60% of all cases is unsatisfactory. An area of particular concern is the very wide distribution of responses in both groups.

The improvement in response to stimulation may have resulted from the use of Ficoll–Triosil at a higher density, since PMNs (presumably effete) and clumped erythrocytes were found to a significant extent in the lower layer and could have contaminated the earlier lower-density preparations.

Our failure to confirm the existence of the spectrum described by Dr Cercek raises several points for speculation. Fig. 3 indicates the rate of build-up of intracellular fluorescein, reaching $3 \times 10^{-9}$M during a typical SCM test. The actual lymphocyte cell volume, however, is of the order of 0·1 $\mu$l per 3 ml of substrate, so the cellular concentration would be approximately $10^{-5}$M, which accounts for the observed red shift in the emission spectrum. It also supports the observation (Udkoff & Norman, 1979; Balding *et al.*, 1980; Hashimoto *et al.*, 1979) that there is a dependence of polarization on intracellular fluorescein concentration, which we confirm (Fig. 3) by the decreasing value of p with increasing time. We also confirm that whilst the fluorescein absorption peak (491 nm) is unaffected by concentrations up to $10^{-2}$M, a significant red shift (up to 505 nm) is produced when the dye is bound by protein. All these effects help to compound the difficulties in accurately quantifying the probe, and perhaps suggest that the measurement of the SCM phenomenon depends on the involvement of a series of artefacts, the balance of which may be altered by the state of health of the donor.

The purpose-built device for directly measuring p was criticized on the basis of the existence of the emission polarization peak at 510 nm, which specifically disappears when the cell is stimulated, and forms the basis of the SCM test (Cercek *et al.*, 1978). This suggested that only very narrow bandwidth filters or gratings could be used to observe the change. Indeed, the sole invalidating argument against this approach rests on the existence of an emission-polarization spectrum which remains unconfirmed.

The reasoning behind the design and construction of this inexpensive instrument was as follows: most of the energy from the Zinc lamp is concentrated into 3 emission lines at 468, 471 and 480 nm, which lie within the absorption spectrum of fluorescein. The use of parallel light from this source overcomes the objection to the extremely low source radiance. The advantage of having simultaneous parallel and perpendicular analyses of fluorescence is in the avoidance of the need to change the axis of the analyser at frequent intervals, which is necessary on single-photomultiplier devices, and it also means that the signals can be processed electronically to monitor polarization continuously. Kinetic studies suggested that a valid measure of cellular polarization could be obtained during the initial stages of the reaction, whilst the background fluorescence is minimal. If so, this would avoid the need for mechanical filtration of the cells which was considered by us to be the weakest and yet most important step in the SCM test as described by the Cerceks. Chromium-labelling experiments by us suggested that lysis of cells during Millipore filtration could be as much as 20%, and this would raise the value of p by lowering the cellular contribution to the apparent total.

In conclusion, therefore, we have been unable to achieve satisfactory distinction between lymphocyte samples from healthy donors or cancer patients using the SCM test, either as originally described or as subsequently modified.

This work was supported by a grant from the Cancer Research Campaign and the Medical Research Council.

### REFERENCES

BAGSHAWE, K. D. (1977) Workshop on macrophage electrophoretic mobility (MEM) and structuredness of cytoplasmic matrix (SCM) tests. *Br. J. Cancer*, **35**, 701.

BALDING, P., LIGHT, P. A. & PREECE, A. W. (1980) Response of human lymphocytes to PHA and tumour-associated antigens as detected by fluorescence polarization, *Br. J. Cancer*, **41**, 73.

BIOZZI, G., STIFFEL, C., MOUTON, D., BOUTHILLIER, Y. & DECREUSEFOND, C. (1968) A kinetic study of antibody producing cells in the spleen of mice immunized intravenously with sheep erythrocytes. *Immunology*, **14**, 7.

CERCEK, L. & CERCEK, B. (1977) Application of the phenomenon of changes in the structuredness of cytoplasmic matrix (SCM) in the diagnosis of malignant disorders: A review. *Eur. J. Cancer*, **13**, 903.

CERCEK, L., CERCEK, B. & FRANKLIN, C. I. V. (1974) Biophysical differentiation between lymphocytes from healthy donors, patients with malignant diseases and other disorders. *Br. J. Cancer*, **29**, 345.

CERCEK, L., CERCEK, B. & OCKEY, C. H. (1978) Fluorescein excitation and emission polarization spectra in living cells. *Biophys. J.*, **23**, 395.

DEIBLER, G. E., MARTENSON, R. E. & KIES, M. W. (1972) Large scale preparation of myelin basic protein from central nervous tissue of several mammalian species. *Prep. Biochem.*, **2**, 139.

HASHIMOTO, Y., TAKAKU, F. & YAMANAKA, T. (1979) Changes in structuredness of cytoplasmic matrix in single stimulated lymphocytes from healthy donors and patients with non-malignant and malignant diseases. *Br. J. Cancer*, **40**, 156.

PREECE, A. W., LIGHT, P. A. & BALDING, P. (1978) Fluorochromasia and fluorescence polarization in lymphoid cells. *Br. J. Cancer*, **38**, 197.

UDKOFF, R. & NORMAN, A. (1979) Polarization of fluorescein fluorescence in single cells. *J. Histochem. Cytochem.*, **27**, 49.


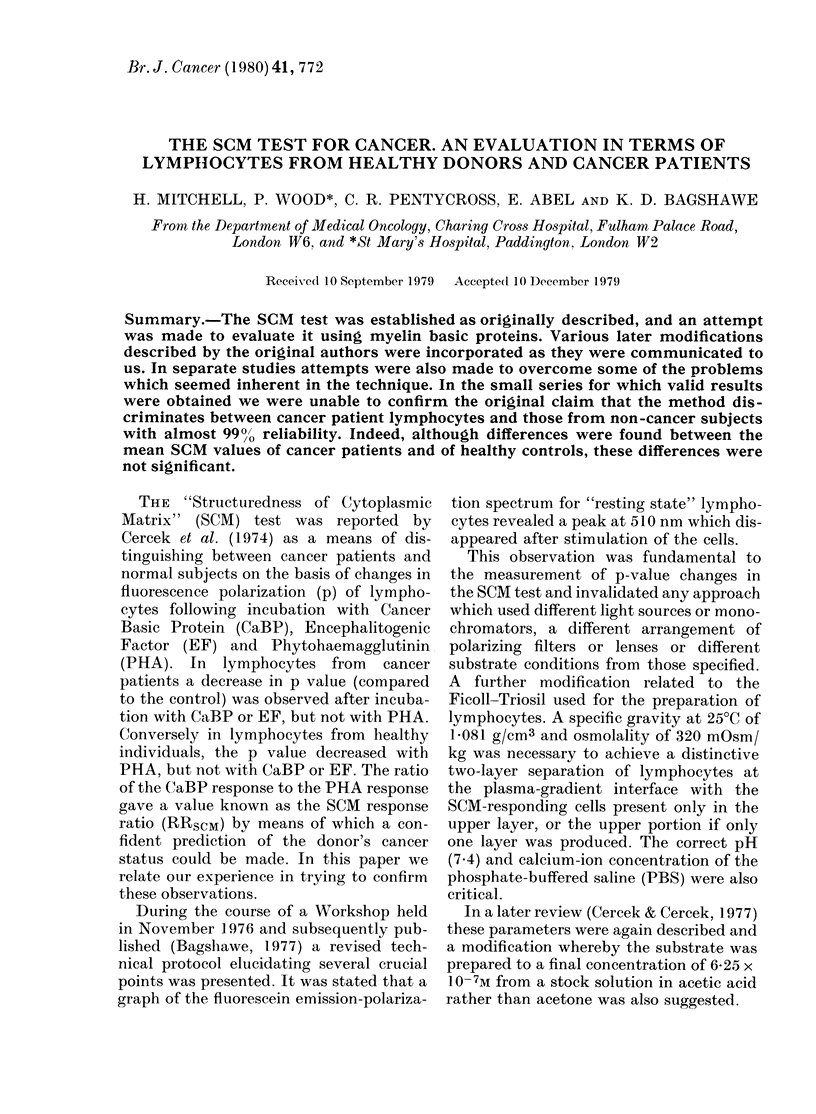

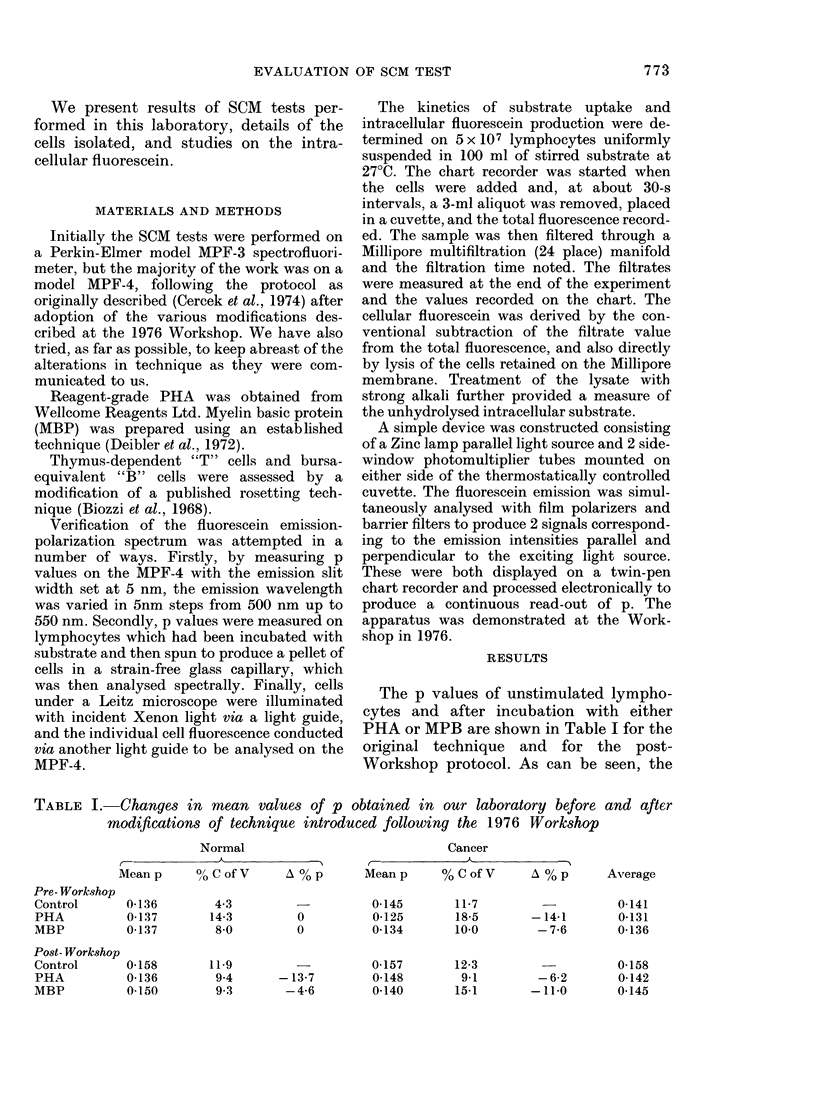

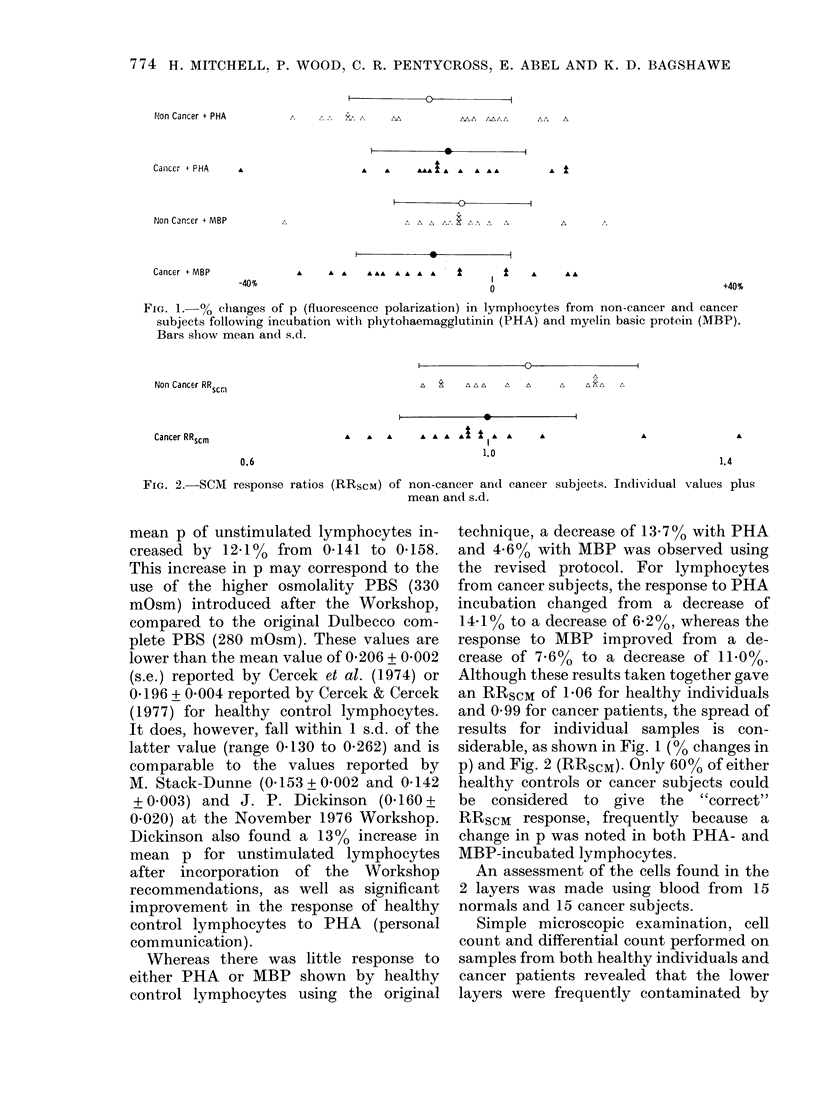

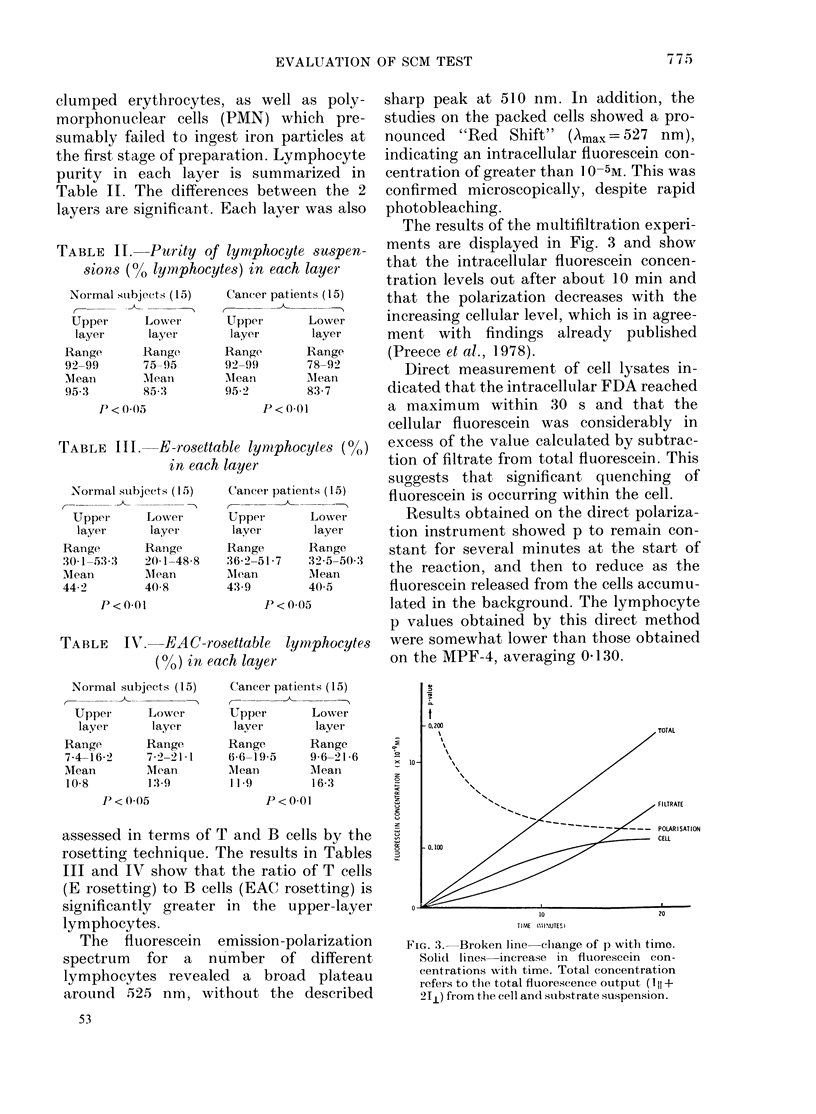

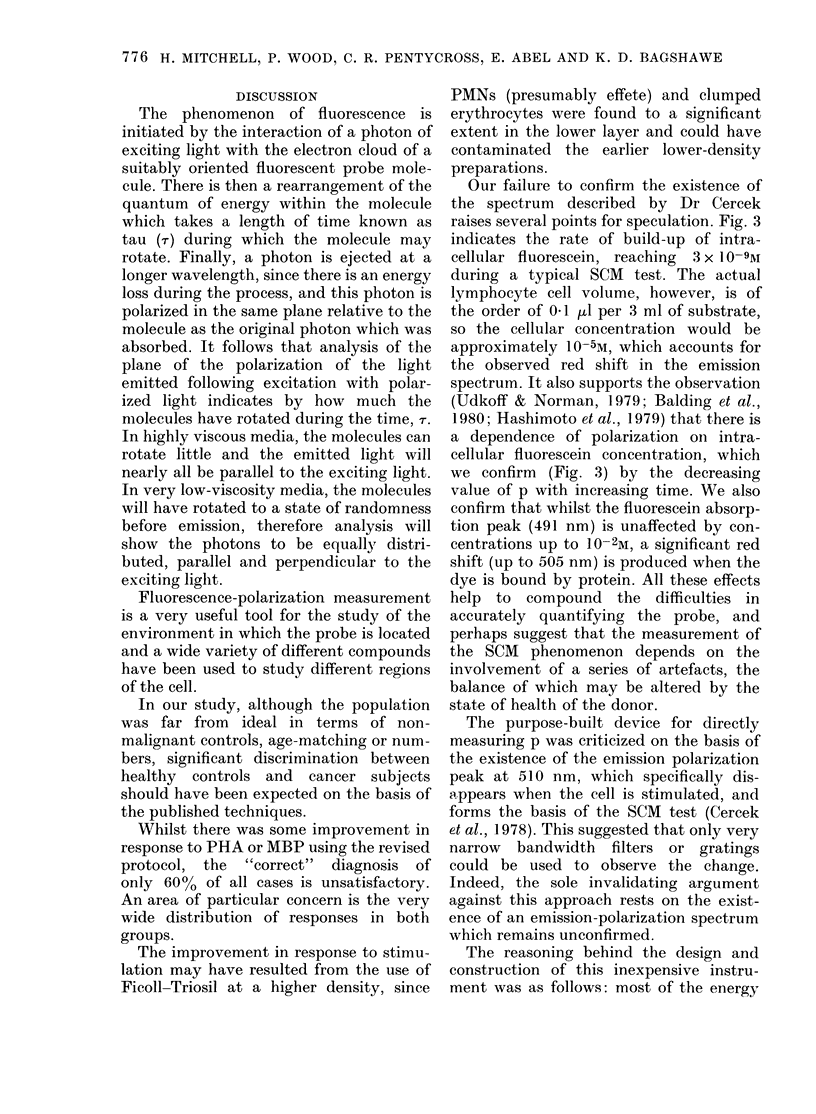

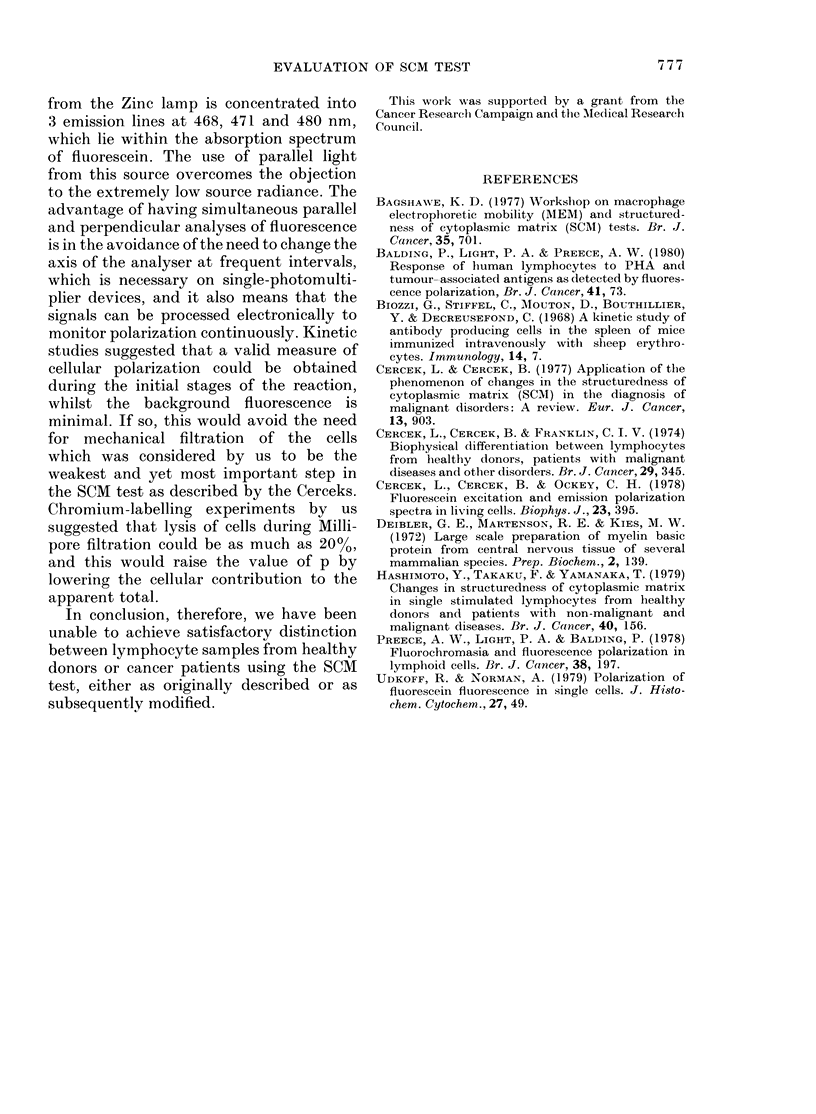


## References

[OCR_00679] Balding P., Light P. A., Preece A. W. (1980). Response of human lymphocytes to PHA and tumour-associated antigens as detected by fluorescence polarization.. Br J Cancer.

[OCR_00685] Biozzi G., Stiffel C., Mouton D., Bouthillier Y., Decreusefond C. (1968). A kinetic study of antibody producing cells in the spleen of mice immunized intravenously with sheep erythrocytes.. Immunology.

[OCR_00692] Cercek L., Cercek B. (1977). Application of the phenomenon of changes in the structuredness of cytoplasmic matrix (SCM) in the diagnosis of malignant disorders: a review.. Eur J Cancer.

[OCR_00699] Cercek L., Cercek B., Franklin C. I. (1974). Biophysical differentiation between lymphocytes from healthy donors, patients with malignant diseases and other disorders.. Br J Cancer.

[OCR_00704] Cercek L., Cercek B., Ockey C. H. (1978). Fluorescein excitation and emission polarization spectra in living cells: changes during the cell cycle.. Biophys J.

[OCR_00709] Deibler G. E., Martenson R. E., Kies M. W. (1972). Large scale preparation of myelin basic protein from central nervous tissue of several mammalian species.. Prep Biochem.

[OCR_00715] Hashimoto Y., Takaku F., Yamanaka T. (1979). Changes in structuredness of cytoplasmic matrix in single stimulated lymphocytes from healthy donors and patients with non-malignant and malignant diseases.. Br J Cancer.

[OCR_00727] Udkoff R., Norman A. (1979). Polarization of fluorescein fluorescence in single cells.. J Histochem Cytochem.

